# Association between the triglyceride–glucose index and pain prognosis after interventional treatment for herpes zoster-associated neuralgia

**DOI:** 10.7717/peerj.21922

**Published:** 2026-11-09

**Authors:** Zhenxiu Liu, Jvming Mao, Huichan Xv, Panqi Wang, Dongkui Gong

**Affiliations:** 1 Department of Anesthesiology and Pain, The First Affiliated Hospital of Soochow University, Suzhou, Jiangsu, China; 2 Department of Urology, The First Affiliated Hospital of Soochow University, Suzhou, Jiangsu, China

**Keywords:** Triglyceride–glucose index, Herpes zoster-associated neuralgia, Interventional pain management, Pain prognosis, Insulin resistance

## Abstract

**Background:**

Pain outcomes after interventional treatment for herpes zoster-associated neuralgia (HZAN) vary substantially, and reliable prognostic markers remain limited. The triglyceride–glucose (TyG) index is a simple surrogate marker of insulin resistance, but its association with pain prognosis after interventional procedures for HZAN is unknown.

**Methods:**

In this retrospective study, patients with HZAN who underwent interventional pain procedures were included. The TyG index was calculated from fasting triglyceride and glucose levels and analyzed as both a continuous variable and by quartiles. Poor pain prognosis at 3 months after discharge was defined using a composite outcome reflecting clinically meaningful pain persistence and impact. Multivariable logistic regression models with incremental adjustment were constructed. To explore potential dose–response associations, restricted cubic spline models were constructed. In addition, prespecified subgroup analyses were conducted to explore potential effect modification across clinical subgroups.

**Results:**

Among 345 patients, 86 (24.9%) met the composite endpoint for poor pain prognosis. After adjustment for clinically relevant covariates, each one-unit increase in TyG was associated with greater odds of poor pain prognosis (adjusted odds ratio (OR) 1.99, 95% confidence interval (CI) [1.25–3.18]). The adjusted OR for the highest *versus* lowest TyG quartile was 2.62 (95% CI [1.19–5.77]). The sensitivity analysis using a numeric rating scale (NRS) ≥4 at 3 months as the sole outcome yielded a similar association (adjusted OR 1.87, 95% CI [1.15–3.05]). Restricted cubic spline analysis found no evidence of nonlinearity, and subgroup analyses provided no statistically significant evidence of effect modification.

**Conclusions:**

The TyG index demonstrates an independent association with pain prognosis after interventional treatment for HZAN, with a linear dose–response relationship. These findings support further investigation of TyG as a metabolic marker associated with pain prognosis, but do not establish its value for individual risk prediction.

## Introduction

Interventional pain management has become an important therapeutic option for patients with herpes zoster-associated neuralgia (HZAN), particularly in cases refractory to pharmacological therapy (Chen et al., 2022). Interventional procedures such as pulsed radiofrequency and spinal cord stimulation have demonstrated efficacy in alleviating HZAN when oral analgesics are insufficient, with both techniques widely reported in clinical series of acute and postherpetic stages of herpes zoster (HZ)-related pain (Wang et al., 2023; Rui et al., 2024). At our center, a standardized interventional strategy has been established, comprising three main techniques: short-term nerve electrical stimulation (NES), radiofrequency thermocoagulation (RF-TC), and pulsed radiofrequency (PRF) (Yuan et al., 2024). Among these, RF-TC and PRF are collectively referred to as radiofrequency (RF)-based interventions, as both rely on radiofrequency energy delivery to modulate nociceptive transmission through targeted application to dorsal root ganglia or peripheral sensory nerves (Guo et al., 2025). However, despite technically appropriate interventions, pain outcomes vary considerably between individuals, with a proportion of patients continuing to experience clinically meaningful pain.

Yet response to these interventional procedures is heterogeneous. Some patients improve quickly and sustain relief, while others continue to report clinically significant pain despite technically appropriate intervention. That variability is not fully explained by the usual clinical predictors. Prior evidence has consistently linked factors such as older age, greater acute pain severity, and comorbidities (including diabetes) with increased risk of postherpetic neuralgia (PHN), underscoring that host factors matter (Forbes et al., 2016). Still, readily available metabolic biomarkers have rarely been incorporated into prognostic evaluation after interventional pain management.

Based on fasting triglyceride and glucose measurements, the triglyceride–glucose (TyG) index has been proposed as a convenient and reproducible indicator reflecting underlying insulin resistance. Large prospective data indicate that higher TyG levels track with future cardiometabolic outcomes, consistent with TyG capturing adverse metabolic physiology relevant to microvascular and neuroinflammatory processes. Mechanistically, insulin resistance and related metabolic stressors are implicated in pathways that can plausibly hinder neural recovery and sustain neuropathic pain, as illustrated by recent high-impact reviews in painful diabetic neuropathy (Eid et al., 2023). These observations provide a rationale for examining the association between TyG and pain prognosis after interventional treatment for HZAN.

To address this gap, this retrospective analysis was performed to investigate the relationship between the TyG index and pain prognosis at 3 months after interventional procedures in patients with HZAN. We evaluated TyG as both a continuous measure and by quartiles, assessed robustness across incrementally adjusted regression models, explored dose–response shape using RCS analysis, and explored potential effect modification across clinical subgroups. Our primary objective was to examine whether TyG was associated with subsequent pain prognosis after adjustment for clinically relevant covariates.

## Materials and Methods

### Study design and population selection

In this retrospective observational investigation, 384 consecutive patients treated in the Department of Anesthesiology and Pain at the First Affiliated Hospital of Soochow University between January 1, 2022, and May 30, 2024, were assessed for eligibility. The study was approved by the Ethics Committee of the First Affiliated Hospital of Soochow University (Approval No. 2025433). The requirement for informed consent was waived by the committee because of the retrospective nature of the study. The detailed process of participant enrollment is presented in Fig. 1. Eligible participants met the following inclusion criteria: (a) a documented history of HZAN of any duration, and (b) undergoing their first inpatient interventional procedure. Exclusion criteria were as follows: (a) age below 20 years; (b) incomplete data regarding the TyG index or pain prognosis at 3 months after discharge; and (c) the presence of other pain-related disorders that could interfere with the assessment of zoster-associated neuralgia. After implementation of the prespecified inclusion and exclusion criteria, 345 individuals were included in the final analytical cohort.

### Assessment of covariates

Demographic data and clinical variables were retrospectively extracted from inpatient medical records. Collected information included age, gender, body weight and height, disease duration (days), pain score at admission (numeric rating scale, NRS) (Bijur, Latimer & Gallagher, 2003), pain location (head/face, trunk, or limbs), type of interventional procedure (PRF, RF-TC, and NES), and comorbidities such as cancer or diabetes mellitus (DM). Disease duration was categorized as <30 days, 30 to <90 days, or ≥90 days. Body mass index (BMI) was derived by dividing body weight (kg) by the square of height (m^2^). Laboratory parameters, including fasting glucose, triglycerides, red blood cell count (RBC), white blood cell count (WBC), lymphocyte percentage (LN), and total protein (TP), were performed at the hospital’s central laboratory.

### Assessment of TyG index

The TyG index was computed according to a standard logarithmic formula based on fasting triglyceride and glucose concentrations: ln (fasting triglycerides (mg/dL) × fasting plasma glucose (mg/dL)/2). For analytical purposes, individuals were stratified into quartile groups (Q1–Q4), with Q1 quartile serving as the reference.

**Figure 1:**
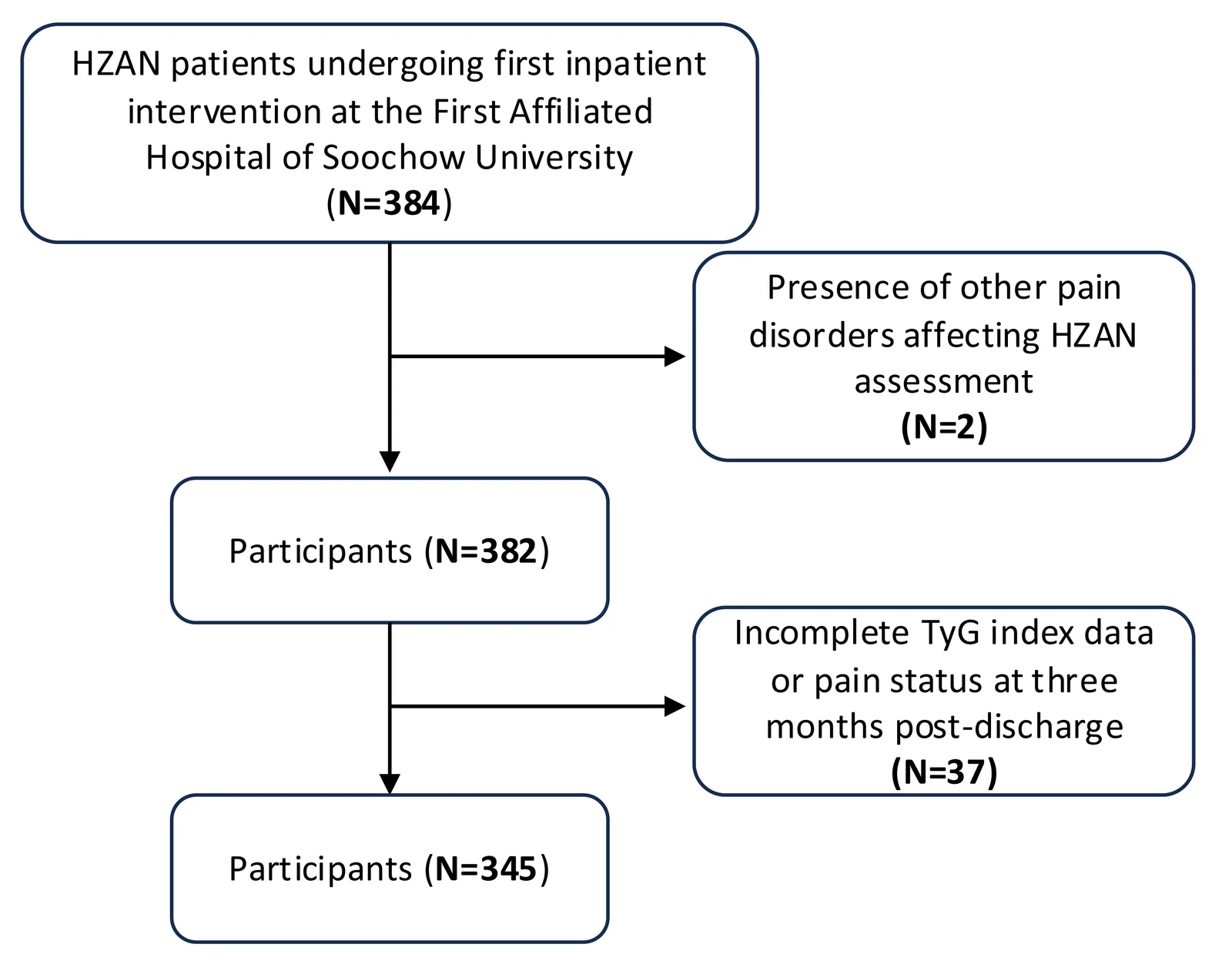
Flowchart depicting the participants’ selection.

### Ascertainment of prognosis in HZAN patients

Patient outcomes were evaluated at 3 months post-discharge through standardized, structured interviews or telephone follow-up. In line with previously established criteria (Watson et al., 1991), a poor prognosis was defined as the occurrence of any of the following at the 3rd month end-point: (1) moderate to severe pain, as reflected by a NRS score ≥4 (0–10 scale) (72 persons); (2) pain-related insomnia, including nocturnal awakenings or difficulty initiating sleep (six persons); (3) clinically significant pain-associated depression or anxiety necessitating pharmacologic intervention (one person); (4) requirement for repeated or multiple interventional pain procedures (four persons); or (5) ongoing analgesic therapy with oral oxycodone, oxcarbazepine, or pregabalin at a total daily dose >300 mg (14 persons). Patients fulfilling any of these criteria were categorized as having a poor prognosis. Among the 345 patients, these components occurred in 72 (20.9%), 6 (1.7%), 1 (0.3%), 4 (1.2%), and 14 (4.1%) patients, respectively. Because individual patients could meet more than one criterion, the component counts were not mutually exclusive and exceeded the 86 patients classified as having poor prognosis.

### Statistical analysis

Baseline characteristics were described across quartiles of the TyG index. Continuous variables were summarized as either means with standard deviations or medians with interquartile ranges, whereas categorical data were summarized as frequencies and proportions. Differences among TyG quartile groups were evaluated using one-way analysis of variance (ANOVA) or the Kruskal-Wallis test for continuous variables, and the *χ*^2^ test or Fisher’s exact test for categorical variables.

The sample size was determined by the available retrospective cohort, and no formal *a priori* sample-size calculation was performed. The analysis included 345 eligible patients, with 86 composite outcome events. The number of events was considered when limiting model complexity. The fully adjusted models contained 10 predictor parameters when TyG was modeled continuously and 12 when modeled in quartiles, excluding the intercept. These corresponded to 8.6 and 7.2 events per parameter, respectively. These ratios were used to describe model complexity relative to the available data, rather than as definitive evidence of model adequacy.

Logistic regression was used to examine associations with poor pain prognosis at 3 months after discharge. TyG was modeled continuously, per one-unit increase, and in quartiles, with Q1 as the reference. Covariate selection combined clinical relevance with univariable findings. Disease duration and baseline NRS score were included based on their clinical relevance and observed associations; age, gender, BMI, DM, and intervention type were retained irrespective of their univariable *P* values. Model 1 was unadjusted; Model 2 adjusted for disease duration and baseline NRS score; and Model 3 additionally adjusted for age, gender, BMI, DM, and intervention type. Results were reported as odds ratios (ORs) with 95% confidence intervals (CIs).

A sensitivity analysis defined the outcome solely as NRS ≥4 at 3 months and applied the same models to all 345 patients regardless of analgesic use. With 72 events, the fully adjusted continuous and quartile models had 7.2 and 6.0 events per parameter, respectively. Frequencies of individual composite-outcome components were reported with overlap permitted.

Restricted cubic spline (RCS) logistic regression was used to explore the shape of the association between TyG and poor pain prognosis, with adjustment for the Model 3 covariates. Splines were fitted using four knots located at 5th, 35th, 65th and 95th percentiles, with the median TyG value as the reference. Tests of the overall association and nonlinearity were reported.

Exploratory subgroup analyses were conducted by age, intervention type, disease duration, baseline NRS score, pain location, and DM, with TyG modeled continuously. Within each stratum, logistic regression models were adjusted for gender, BMI, age, DM, intervention type, disease duration, and baseline NRS score, excluding the corresponding stratifying variable when it belonged to this adjustment set. Models stratified by pain location retained all seven covariates. Effect modification was assessed in the full cohort using models that included TyG, the subgroup variable, their interaction term(s), and the remaining adjustment covariates. Nonsignificant interaction tests were not interpreted as evidence of equivalent associations across subgroups.

Model discrimination was explored using receiver operating characteristic curves and areas under the curve with 95% confidence intervals. These estimates describe apparent discrimination in the study cohort and were not interpreted as validation of a clinical prediction model. Multicollinearity and model-fit diagnostics are reported in the Supplementary Materials. All analyses were performed using R version 4.2.2 (R Core Team, 2022). Tests were two-sided, with *P* < 0.05 considered statistically significant.

## Results

### Baseline characteristics of the study population

As shown in Table 1, individuals older than 65 years accounted for more than half of the cohort in each TyG category, and no statistically significant variation in age distribution was observed across the quartile groups (*P* = 0.083), indicating comparable age structures among the groups. BMI increased progressively with higher TyG quartiles (*P* = 0.009), whereas gender distribution was comparable across groups (*P* = 0.769). The proportion of patients with DM increased progressively from Q1 to Q4 (8.1% to 29.9%, *P* = 0.001), whereas cancer prevalence showed no significant difference across TyG quartiles. Higher TyG quartiles were associated with higher WBC and RBC (*P* = 0.002 and *P* = 0.043, respectively), whereas LN and TP levels were similar across groups.

**Table 1:** Patient demographics and baseline characteristics.

**Characteristic**	**TyG**				***P*-value**
	**Q1 ≤ 8.34** ***N* = 86**	**8.34 < Q2 ≤ 8.68** ***N* = 86**	**8.68 < Q3 ≤ 9.17** ***N* = 86**	**Q4 > 9.17** ***N* = 87**	
Age, *n* (%)					0.083^a^
<=65	27 (31.4%)	23 (26.7%)	34 (39.5%)	38 (43.7%)	
>65	59 (68.6%)	63 (73.3%)	52 (60.5%)	49 (56.3%)	
BMI, kg/m2, Mean ± SD	22.5 ± 3.0	23.1 ± 3.4	23.5 ± 2.9	24.0 ± 3.1	0.009^b^
Gender, *n* (%)					0.769^a^
Male	44 (51.2%)	43 (50.0%)	41 (47.7%)	38 (43.7%)	
Female	42 (48.8%)	43 (50.0%)	45 (52.3%)	49 (56.3%)	
Diabetes, *n* (%)					0.001^a^
No	79 (91.9%)	74 (86.0%)	66 (76.7%)	61 (70.1%)	
Yes	7 (8.1%)	12 (14.0%)	20 (23.3%)	26 (29.9%)	
Cancer, *n* (%)					0.325^a^
No	73 (84.9%)	79 (91.9%)	73 (84.9%)	79 (90.8%)	
Yes	13 (15.1%)	7 (8.1%)	13 (15.1%)	8 (9.2%)	
WBC, Median (Q1, Q3)	5.21 (4.29, 6.28)	5.85 (4.89, 7.10)	6.39 (5.31, 7.41)	5.88 (5.29, 7.33)	0.002^c^
RBC, Median (Q1, Q3)	4.07 (3.74, 4.39)	4.21 (3.83, 4.50)	4.28 (4.01, 4.56)	4.21 (3.93, 4.59)	0.043^c^
LN, %, Median (Q1, Q3)	29 (19, 35)	30 (21, 35)	28 (20, 34)	29 (22, 37)	0.830^c^
TP, Median (Q1, Q3)	63.4 (60.1, 68.2)	63.9 (60.9, 67.7)	65.2 (61.5, 69.1)	65.4 (61.5, 68.0)	0.354^c^
Duration, days, *n* (%)					0.003^a^
<30	14 (16.3%)	12 (14.0%)	9 (10.5%)	27 (31.0%)	
>=30 & <90	47 (54.7%)	44 (51.2%)	55 (64.0%)	32 (36.8%)	
>=90	25 (29.1%)	30 (34.9%)	22 (25.6%)	28 (32.2%)	
Location, *n* (%)					0.700^a^
Trunk	45 (52.3%)	45 (52.3%)	48 (55.8%)	49 (56.3%)	
Head & Face	16 (18.6%)	13 (15.1%)	14 (16.3%)	8 (9.2%)	
Limbs	25 (29.1%)	28 (32.6%)	24 (27.9%)	30 (34.5%)	
NRS score, *n* (%)					0.730^a^
<7	25 (29.1%)	26 (30.2%)	23 (26.7%)	30 (34.5%)	
>=7	61 (70.9%)	60 (69.8%)	63 (73.3%)	57 (65.5%)	
Interventional procedure, *n* (%)					0.668^a^
PRF	46 (53.5%)	49 (57.0%)	48 (55.8%)	54 (62.1%)	
RF-TC	19 (22.1%)	16 (18.6%)	23 (26.7%)	18 (20.7%)	
NES	21 (24.4%)	21 (24.4%)	15 (17.4%)	15 (17.2%)	
Pain prognosis, *n* (%)					0.048^a^
Favorable	72 (83.7%)	68 (79.1%)	60 (69.8%)	59 (67.8%)	
Poor	14 (16.3%)	18 (20.9%)	26 (30.2%)	28 (32.2%)	

**Notes.**

a Pearson’s Chi-squared test;

b One-way analysis of means;

c Kruskal-Wallis rank sum test.

RBC red blood cell WBC white blood cell L Lymphocyte NRS numerical rating scale RF radiofrequency NES nerve electrical stimulation

Disease duration differed significantly across TyG quartiles (*P* = 0.003), with a higher proportion of patients in the highest TyG quartile presenting within 30 days of symptom onset. Pain location, baseline pain intensity assessed by the numeric rating scale, and type of interventional procedure were comparable among quartiles. At 3-month follow-up, the proportion of patients with poor pain prognosis increased progressively across TyG quartiles (16.3% *vs.* 32.2%, *P* = 0.048).

### Association between TyG index and pain prognosis after interventional procedures

Univariable and multivariable logistic regression analyses were performed to evaluate factors associated with poor pain prognosis at 3 months after interventional treatment (Fig. 2 and Table S1). TyG was retained as the primary exposure. Disease duration and baseline NRS score were included based on their clinical relevance and observed associations with the outcome in univariable analyses. Age, gender, BMI, DM, and intervention type were additionally included as clinically relevant potential confounders, regardless of their univariable *P* values. In univariable analysis, a higher TyG index was significantly associated with an increased risk of poor pain prognosis (OR 1.77, 95% CI [1.17–2.67]; *P* = 0.007). This association remained robust after adjustment for potential confounders, including age, BMI, gender, diabetes, disease duration, pain intensity, and type of interventional procedure (adjusted OR 1.99, 95% CI [1.25–3.18]; *P* = 0.004).

**Figure 2:**
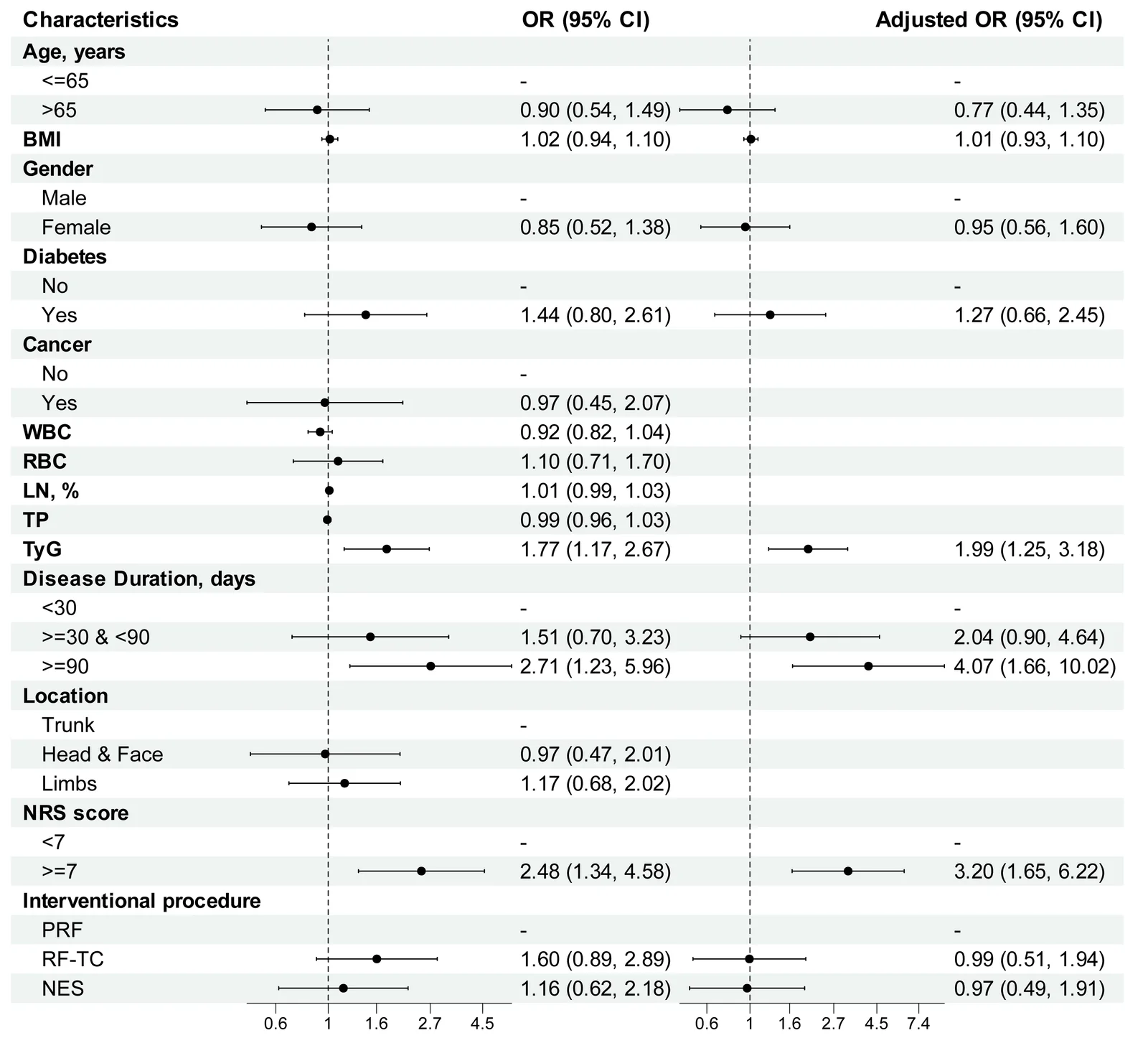
Univariable and multivariable logistic regression analyses of factors associated with poor pain prognosis at 3 months after interventional treatment for herpes zoster-associated neuralgia. The forest plot presents odds ratios (OR) and adjusted odds ratios (aOR) with 95% confidence intervals (CIs). The multivariable model included the TyG index and was adjusted for age, gender, BMI, diabetes, disease duration, baseline NRS score, and intervention type. ORs represent the change in odds per one-unit increase; reference categories for categorical variables are indicated by dashes. Adjusted estimates are presented for variables included in the clinically defined core adjustment set.

Disease duration was also independently associated with pain prognosis. Compared with patients presenting within 30 days of symptom onset, those with a disease duration of ≥90 days had a significantly higher risk of poor pain outcomes in both univariable (OR 2.71, 95% CI [1.23–5.96]; *P* = 0.013) and multivariable analyses (adjusted OR 4.07, 95% CI [1.66–10.02]; *P* = 0.002). A disease duration of 30 to <90 days showed a trend toward worse prognosis in the multivariable model, although this did not reach statistical significance (adjusted OR 2.04, 95% CI [0.90–4.64]; *P* = 0.089).

Higher baseline pain intensity was also associated with greater odds of poor pain prognosis. Patients with a baseline NRS score ≥7 had a significantly increased likelihood of poor pain prognosis compared with those with NRS <7, both in univariable analysis (OR 2.48, 95% CI [1.34–4.58]; *P* = 0.004) and after multivariable adjustment (adjusted OR 3.20, 95% CI [1.65–6.22]; *P* < 0.001).

In contrast, age, gender, BMI, diabetes, and type of interventional procedure were not independently associated with pain prognosis in the multivariable model.

### Association between TyG index and pain prognosis across multiple regression models

To further assess the robustness of the relationship between the TyG index and pain prognosis, three logistic regression models incorporating progressively more covariates were developed, as detailed in Table 2. When modeled as a continuous variable, the TyG index was significantly associated with poor pain prognosis in the unadjusted model (Model 1: OR 1.77, 95% CI [1.17–2.67]; *P* = 0.007). This association remained consistent after adjustment for disease duration, baseline pain intensity (Model 2: OR 2.13, 95% CI [1.36–3.34]; *P* < 0.001), and remained associated after further adjustment for disease duration, baseline pain intensity, age, gender, BMI, DM, and intervention type (Model 3: OR 1.99, 95% CI [1.25–3.18]; *P* = 0.004).

**Table 2:** Association between TyG and pain prognosis with multiple regression models.

**Characteristic**	**Model 1**			**Model 2**			**Model 3**		
	**OR**	**95% CI**	***P*-value**	**OR**	**95% CI**	***P*-value**	**OR**	**95% CI**	***P*-value**
TyG (continuous)	1.77	1.17, 2.67	0.007	2.13	1.36, 3.34	<0.001	1.99	1.25, 3.18	0.004
TyG									
Q1	–	–		–	–		–	–	
Q2	1.36	0.63, 2.95	0.434	1.32	0.60, 2.90	0.498	1.31	0.59, 2.90	0.514
Q3	2.23	1.07, 4.65	0.032	2.21	1.04, 4.72	0.040	2.06	0.95, 4.48	0.069
Q4	2.44	1.18, 5.05	0.016	2.87	1.34, 6.15	0.007	2.62	1.19, 5.77	0.017
*P* for trend			0.007			0.002			0.008

**Notes.**

Model 1: no covariates were adjusted.

Model 2: adjusted for Disease Duration, and baseline NRS score.

Model 3: adjusted for Gender, BMI, Age, Diabetes, Interventional procedure, Disease Duration, and baseline NRS score.

Moreover, higher TyG index levels were consistently associated with an elevated likelihood of unfavorable pain outcomes. Specifically, individuals in the top TyG quartile (Q4) had a markedly greater risk of poor pain prognosis than those in the lowest quartile (Q1) in all three regression models. In the fully adjusted model, the adjusted OR for Q4 *versus* Q1 was 2.62 (95% CI [1.19–5.77]; *P* = 0.017). A clear gradient effect was observed across TyG quartiles, with a significant trend toward increasing risk of poor prognosis with higher TyG levels (*P* for trend = 0.008 in Model 3). Model diagnostics and multicollinearity assessments are provided in Tables S2–S3.

Exploratory receiver operating characteristic (ROC) analysis yielded an AUC of 0.595 (95% CI [0.527–0.663]) for Model 1 and 0.703 (95% CI [0.642–0.764]) for both Models 2 and 3 (Fig. 3). These estimates describe apparent discrimination within the study cohort and do not establish validated predictive performance or clinical utility.

**Figure 3:**
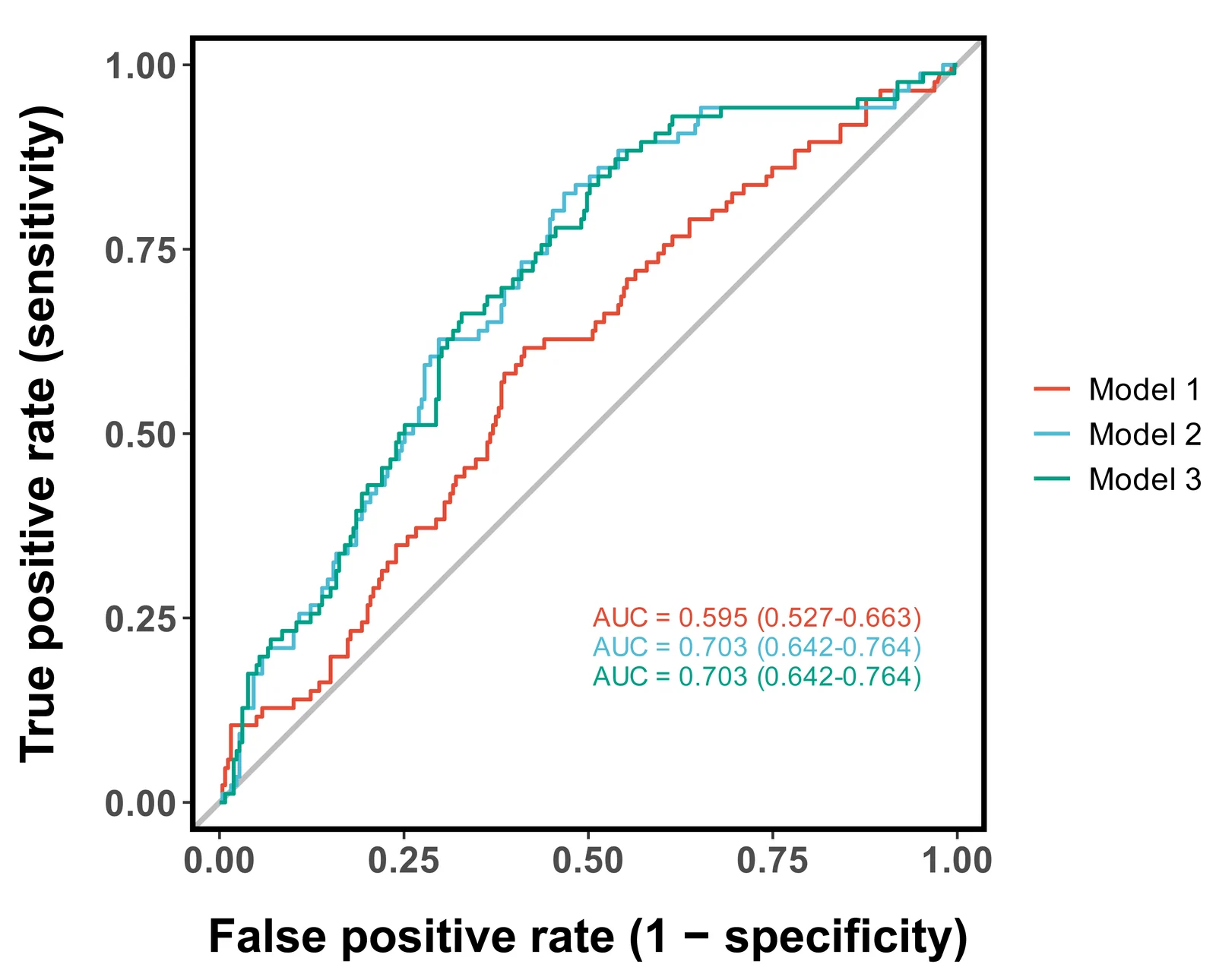
Exploratory assessment of apparent discrimination for logistic regression models of poor pain prognosis at 3 months after discharge. Model 1 was unadjusted; Model 2 was adjusted for disease duration and baseline NRS score; and Model 3 additionally included age, gender, BMI, diabetes mellitus, and intervention type. Areas under the curve (AUCs) with 95% confidence intervals are shown. These estimates were obtained from the study cohort without internal or external validation.

### Sensitivity analysis using an NRS score ≥4 as the outcome

To assess the robustness of the findings to the definition of the primary outcome, we performed a sensitivity analysis in which the outcome was defined solely as an NRS score ≥4 at 3 months. This outcome occurred in 72 of the 345 patients (20.9%). When analyzed as a continuous variable, a higher TyG index was associated with greater odds of an NRS score ≥4 in the unadjusted model (OR 1.66, 95% CI [1.07–2.56]; *P* = 0.023). The association persisted after adjustment for disease duration and NRS score (adjusted OR 1.97, 95% CI [1.23–3.14]; *P* = 0.005) and after further adjustment for age, gender, BMI, DM, and intervention type (adjusted OR 1.87, 95% CI [1.15–3.05]; *P* = 0.012). In the fully adjusted quartile analysis, patients in the highest TyG quartile had greater odds of an NRS score ≥4 than those in the lowest quartile (adjusted OR 2.26, 95% CI [1.00–5.09]; *P* = 0.049), with a significant trend across quartiles (*P* for trend = 0.044). These findings were directionally consistent with the primary analysis using the composite outcome (Tables S4 and S5).

### RCS analysis of the TyG and pain prognosis

In order to investigate the potential nonlinear pattern, RCS analysis was performed with adjustment for gender, BMI, age, diabetes, interventional procedure, disease duration, and NRS score. As shown in Fig. 4, the TyG index was positively associated with the odds of poor pain prognosis across its observed range. The overall association between TyG and pain prognosis was statistically significant (*P*-overall = 0.039). In contrast, there was no evidence of a nonlinear relationship (*P*-nonlinear = 0.982), indicating that the association between TyG and pain prognosis was approximately linear on the log-odds scale. Details of the RCS model coefficients and diagnostic statistics are provided in Table S6.

**Figure 4:**
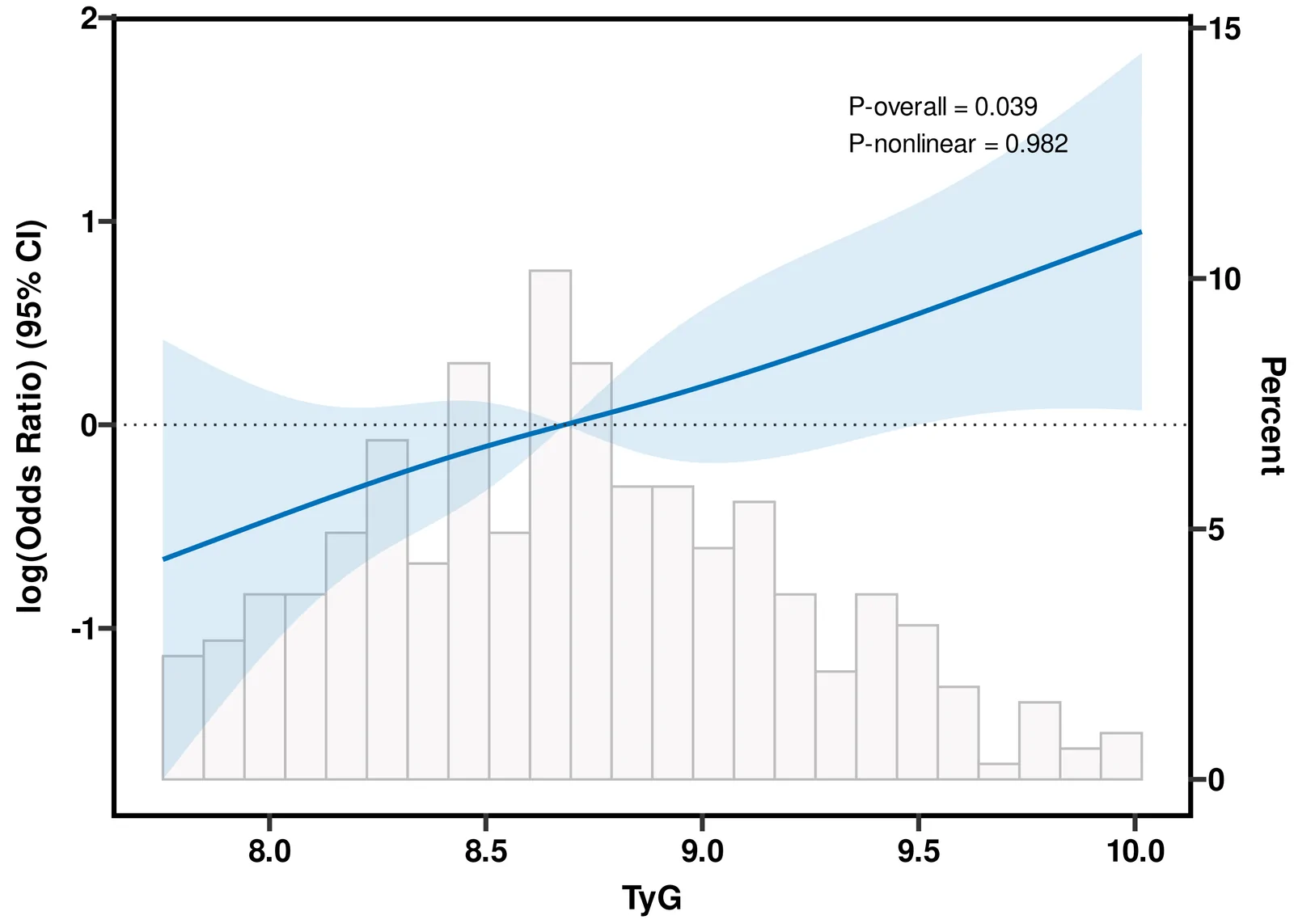
Association between TyG and pain prognosis with the RCS function. Model with four knots located at 5th, 35th, 65th and 95th percentiles. *Y*-axis represents the OR to present pain prognosis for any value of TyG compared to individuals with reference value (50th percentile) of TyG. The RCS model was adjusted for Gender, BMI, Age, Diabetes, Interventional procedure, Disease Duration, and baseline NRS score.

Specifically, the estimated odds of poor pain prognosis increased progressively with higher TyG values, with no apparent threshold or inflection point within the studied range. The confidence intervals widened at the extremes of the TyG distribution, reflecting fewer observations in these regions.

### Subgroup analyses

Subgroup models were adjusted for the core covariates, excluding the stratifying variable when it belonged to the adjustment set. There was no statistically significant evidence of effect modification by age, intervention type, disease duration, baseline NRS score, pain location, or DM (all *P* for interaction >0.05; Fig. S1 and Table S7). With the three intervention categories analyzed separately, the adjusted OR per one-unit increase in TyG was 2.06 (95% CI [1.07–3.95]) for PRF, 1.11 (95% CI [0.47–2.64]) for RF-TC, and 4.65 (95% CI [1.14–18.89]) for NES (*P* for interaction = 0.559). Subgroup estimates varied in magnitude and precision, and the association did not reach statistical significance in every subgroup. The wide confidence intervals in several subgroups warrant cautious interpretation of these exploratory findings.

## Discussion

This retrospective study demonstrated that elevated TyG index values were independently linked to a higher likelihood of poor pain prognosis at 3 months in HZAN patients undergoing interventional pain procedures. This relationship persisted after comprehensive adjustment for demographic characteristics, metabolic comorbidities, disease duration, baseline pain intensity, and type of interventional procedure, and was consistent with multiple regression models with increasing levels of covariate control. Furthermore, RCS analysis demonstrated an approximately linear dose–response relationship between TyG and pain prognosis, with no evidence of a nonlinear threshold effect. The subgroup analyses provided no statistically significant evidence of effect modification, although wide confidence intervals in several subgroups limited conclusions about differences between groups.

Mechanistically, TyG index serves as an inexpensive and commonly applied proxy for insulin resistance (Kurniawan, 2024). Insulin resistance and related metabolic perturbations may plausibly influence zoster-associated neuropathic pain through converging pathways that are also central to other painful neuropathies, including mitochondrial/bioenergetic dysfunction, microvascular injury, oxidative stress, and neuroimmune activation (Eid et al., 2023). In particular, neuroinflammation mediated by glial and immune effectors has emerged as an important contributor to neuropathic pain maintenance; experimental and translational work in diabetic neuropathic pain highlights the role of microglial activation in amplifying nociceptive signaling under hyperglycemic and inflammatory milieus (Wang et al., 2024). Against this background, a higher TyG index may identify patients with a metabolic state less favorable for neural recovery after varicella-zoster virus-related nerve injury, thereby predisposing to persistent pain despite otherwise appropriate interventional management.

Our findings also align with contemporary evidence that metabolic comorbidity—particularly diabetes—is linked to herpes zoster susceptibility and PHN risk. A recent study identified diabetes among conditions associated with increased herpes zoster risk, supporting the broader connection between metabolic disease and zoster-related outcomes (Steinmann et al., 2024). Moreover, a recent meta-analysis of PHN risk factors reported DM and higher acute pain severity among factors associated with PHN, which is consistent with the strong prognostic contribution of baseline pain intensity observed in our cohort (Wang et al., 2025). Importantly, existing zoster pain literature has largely focused on demographic/clinical predictors and treatment timing, whereas metabolic markers that are readily available in routine care have been comparatively understudied. Our findings extend this literature by showing an association between TyG and subsequent pain prognosis after adjustment for demographic and clinical factors.

The association between TyG and pain prognosis warrants further investigation of its potential clinical relevance. TyG can be calculated from routine fasting laboratory measurements and has been used as a surrogate marker of insulin resistance in large populations (Lopez-Jaramillo et al., 2023). However, the present study does not establish its value for individual risk prediction or treatment selection. Although the spline analysis found no evidence of nonlinearity, it did not establish a clinically useful cutoff. Similarly, nonsignificant interaction tests do not demonstrate equivalent associations across subgroups, particularly given the wide confidence intervals in several strata. Prospective studies with appropriate validation are needed to determine whether TyG adds useful prognostic information beyond established clinical factors and can inform patient management.

Several limitations should be acknowledged. To begin with, the retrospective nature of this single-center analysis may predispose the study to selection bias and limits causal inference. The sample size was determined by the available retrospective cohort. Although model complexity was limited to a core adjustment set, the number of outcome events relative to the number of parameters leaves a risk of overfitting and imprecise estimates. This concern is particularly relevant to the quartile, sensitivity, and subgroup analyses. The reported events-per-parameter ratios should therefore not be interpreted as establishing model adequacy. Second, TyG was assessed at a single time point, and we could not evaluate whether changes in metabolic status over time modify pain trajectories. Third, despite adjustment for age, gender, BMI, DM, disease duration, NRS score, and intervention type, residual confounding remains possible, including from antiviral timing, vaccination status, lesion severity, psychosocial factors, and medication adherence.

The composite endpoint captured pain intensity alongside sleep disturbance, psychological symptoms, repeat interventions, and ongoing analgesic requirements. Its heterogeneous components may limit comparability with studies using pain intensity alone and generalizability to settings with different treatment practices. Although NRS ≥4 accounted for most composite events and the pain-only sensitivity analysis yielded a similar association, analgesic use at follow-up may have lowered measured NRS scores. The sensitivity analysis therefore supports the primary finding but does not eliminate the limitations of the composite endpoint.

These findings warrant further investigation of TyG as a metabolic marker of pain prognosis, but do not establish causality or clinical predictive utility. Prospective studies are needed to confirm the association and determine whether TyG provides useful information beyond established clinical factors.

## Ethics statement

The study protocol adhered to the principles outlined in the Declaration of Helsinki and received approval from the Ethics Committee of the First Affiliated Hospital of Soochow University (Approval No. 2025433). Given the retrospective observational design and the use of anonymized data, the requirement for written informed consent was waived by the institutional review board.

##  Supplemental Information

10.7717/peerj.21922/supp-1 Supplemental Information 1 STROBE checklist

10.7717/peerj.21922/supp-2 Supplemental Information 2 Figure S1 & Tables S1-5

10.7717/peerj.21922/supp-3 Data S1 Raw data

10.7717/peerj.21922/supp-4 Supplemental Information 4 Codebook for Categorical Variables

## Additional Information and Declarations

### Data Availability

The following information was supplied regarding data availability: The raw measurements are available in the Supplemental Files.
