# Supplementary material for: Association between the triglyceride–glucose index and pain prognosis after interventional treatment for herpes zoster-associated neuralgia
Source: PeerJ. 2026 Nov 9;14:e21922. doi: 10.7717/peerj.21922 (PMC13757739; doi:10.7717/peerj.21922)
Supplement: Supplementary file 1 [file peerj-14-21922-s004.pdf]

Codebook for Categorical Variables

Variable	Code	Description
Gender	0	Male
Gender	1	Female
Location	0	Trunk
Location	1	Head & Face
Location	2	Limbs
Interventional procedure	1	PRF, pulsed radiofrequency
Interventional procedure	2	RF-TC, radiofrequency thermocoagulation
Interventional procedure	3	NES, short-term nerve electrical stimulation
Interventional procedure_2	1	RF, radiofrequency
Interventional procedure_2	2	NES, short-term nerve electrical stimulation
Duration	0	<30 days
Duration	1	⩾30 & <90 days
Duration	2	⩾90 days
Diabetes	0	No
Diabetes	1	Yes
Cancer	0	No
Cancer	1	Yes
NRS score	0	<7
NRS score	1	⩾7
Age	0	⩽65 years
Age	1	>65 years
Pain prognosis	0	Favorable
Pain prognosis	1	Poor
oNRS score (pain prognosis at 3 months was defined solely as an NRS score ⩾4)	0	Favorable
oNRS score (pain prognosis at 3 months was defined solely as an NRS score ⩾4)	1	Poor
insomnia	1	Yes

depression or anxiety	1	Yes
analgesic therapy	1	Yes
