# Supplementary material for: Association between the triglyceride–glucose index and pain prognosis after interventional treatment for herpes zoster-associated neuralgia
Source: PeerJ. 2026 Nov 9;14:e21922. doi: 10.7717/peerj.21922 (PMC13757739; doi:10.7717/peerj.21922)
Supplement: Supplementary file 3 [file peerj-14-21922-s002.docx]

Supplementary Materials. Figure S1 & Table S1-5

**Figure S1**. Subgroup analyses of the association between TyG index and pain prognosis

Table S1. Univariable and multivariable analysis of influencing factors (Logistic regression)

| **Characteristic** | **Univariable** | | | | | **Multivariable** | | | | |
| --- | --- | --- | --- | --- | --- | --- | --- | --- | --- | --- |
|  | **N** | **Event N** | **OR** | **95% CI** | **p-value** | **N** | **Event N** | **OR** | **95% CI** | **p-value** |
| **Age** |  |  |  |  |  |  |  |  |  |  |
| <=65 | 122 | 32 | — | — |  | 122 | 32 | — | — |  |
| >65 | 223 | 54 | 0.90 | 0.54, 1.49 | 0.679 | 223 | 54 | 0.77 | 0.44, 1.35 | 0.353 |
| **BMI** | 345 | 86 | 1.02 | 0.94, 1.10 | 0.699 | 345 | 86 | 1.01 | 0.93, 1.10 | 0.793 |
| **Gender** |  |  |  |  |  |  |  |  |  |  |
| Male | 166 | 44 | — | — |  | 166 | 44 | — | — |  |
| Female | 179 | 42 | 0.85 | 0.52, 1.38 | 0.514 | 179 | 42 | 0.95 | 0.56, 1.60 | 0.838 |
| **Diabetes** |  |  |  |  |  |  |  |  |  |  |
| No | 280 | 66 | — | — |  | 280 | 66 | — | — |  |
| Yes | 65 | 20 | 1.44 | 0.80, 2.61 | 0.228 | 65 | 20 | 1.27 | 0.66, 2.45 | 0.475 |
| **Cancer** |  |  |  |  |  |  |  |  |  |  |
| No | 304 | 76 | — | — |  |  |  |  |  |  |
| Yes | 41 | 10 | 0.97 | 0.45, 2.07 | 0.932 |  |  |  |  |  |
| **WBC** | 345 | 86 | 0.92 | 0.82, 1.04 | 0.190 |  |  |  |  |  |
| **RBC** | 345 | 86 | 1.10 | 0.71, 1.70 | 0.666 |  |  |  |  |  |
| **LN, %** | 345 | 86 | 1.01 | 0.99, 1.03 | 0.383 |  |  |  |  |  |
| **TP** | 345 | 86 | 0.99 | 0.96, 1.03 | 0.628 |  |  |  |  |  |
| **TyG** | 345 | 86 | 1.77 | 1.17, 2.67 | 0.007 | 345 | 86 | 1.99 | 1.25, 3.18 | 0.004 |
| **Disease Duration, days** |  |  |  |  |  |  |  |  |  |  |
| <30 | 62 | 10 | — | — |  | 62 | 10 | — | — |  |
| >=30 & <90 | 178 | 40 | 1.51 | 0.70, 3.23 | 0.292 | 178 | 40 | 2.04 | 0.90, 4.64 | 0.089 |
| >=90 | 105 | 36 | 2.71 | 1.23, 5.96 | 0.013 | 105 | 36 | 4.07 | 1.66, 10.02 | 0.002 |
| **Location** |  |  |  |  |  |  |  |  |  |  |
| Trunk | 187 | 45 | — | — |  |  |  |  |  |  |
| Head & Face | 51 | 12 | 0.97 | 0.47, 2.01 | 0.937 |  |  |  |  |  |
| Limbs | 107 | 29 | 1.17 | 0.68, 2.02 | 0.564 |  |  |  |  |  |
| **Pre-op NRS score** |  |  |  |  |  |  |  |  |  |  |
| <7 | 104 | 15 | — | — |  | 104 | 15 | — | — |  |
| >=7 | 241 | 71 | 2.48 | 1.34, 4.58 | 0.004 | 241 | 71 | 3.20 | 1.65, 6.22 | <0.001 |
| **Interventional procedure** |  |  |  |  |  |  |  |  |  |  |
| PRF | 197 | 44 | — | — |  | 197 | 44 | — | — |  |
| RF-TC | 76 | 24 | 1.60 | 0.89, 2.89 | 0.115 | 76 | 24 | 0.99 | 0.51, 1.94 | 0.984 |
| NES | 72 | 18 | 1.16 | 0.62, 2.18 | 0.646 | 72 | 18 | 0.97 | 0.49, 1.91 | 0.923 |
| Abbreviations: CI = Confidence Interval, OR = Odds Ratio | | | | | | | | | | |
| Null deviance = 387; Null df = 344; Log-likelihood = -178; AIC = 378; BIC = 420; Deviance = 356; Residual df = 334; No. Obs. = 345 | | | | | | | | | | |

Table S2. Multicollinearity diagnostics of variables included in the multivariable regression models.

**Model I**

None

**Model II**

**Model III**

**Table S3.** Model fit indices for logistic regression models evaluating the association between TyG index and pain prognosis.

**Table S4.** Univariable and multivariable analysis of influencing factors **(For the sensitivity analysis, poor pain prognosis at 3 months was defined solely as an NRS score ≥4)**

| **Characteristic** | **Univariable** | | | | | **Multivariable** | | | | |
| --- | --- | --- | --- | --- | --- | --- | --- | --- | --- | --- |
|  | **N** | **Event N** | **OR** | **95% CI** | **p-value** | **N** | **Event N** | **OR** | **95% CI** | **p-value** |
| **Age** |  |  |  |  |  |  |  |  |  |  |
| <=65 | 122 | 26 | — | — |  | 122 | 26 | — | — |  |
| >65 | 223 | 46 | 0.96 | 0.56, 1.65 | 0.881 | 223 | 46 | 0.84 | 0.46, 1.52 | 0.564 |
| **BMI** | 345 | 72 | 1.01 | 0.93, 1.10 | 0.845 | 345 | 72 | 1.01 | 0.92, 1.10 | 0.887 |
| **Gender** |  |  |  |  |  |  |  |  |  |  |
| Male | 166 | 35 | — | — |  | 166 | 35 | — | — |  |
| Female | 179 | 37 | 0.98 | 0.58, 1.64 | 0.925 | 179 | 37 | 1.11 | 0.64, 1.93 | 0.719 |
| **Diabetes** |  |  |  |  |  |  |  |  |  |  |
| No | 280 | 55 | — | — |  | 280 | 55 | — | — |  |
| Yes | 65 | 17 | 1.45 | 0.77, 2.71 | 0.246 | 65 | 17 | 1.31 | 0.66, 2.60 | 0.440 |
| **Cancer** |  |  |  |  |  |  |  |  |  |  |
| No | 304 | 64 | — | — |  |  |  |  |  |  |
| Yes | 41 | 8 | 0.91 | 0.40, 2.06 | 0.820 |  |  |  |  |  |
| **WBC** | 345 | 72 | 0.91 | 0.80, 1.04 | 0.151 |  |  |  |  |  |
| **RBC** | 345 | 72 | 1.16 | 0.73, 1.85 | 0.534 |  |  |  |  |  |
| **LN, %** | 345 | 72 | 1.01 | 0.99, 1.04 | 0.368 |  |  |  |  |  |
| **TP** | 345 | 72 | 0.98 | 0.94, 1.02 | 0.268 |  |  |  |  |  |
| **TyG** | 345 | 72 | 1.66 | 1.07, 2.56 | 0.023 | 345 | 72 | 1.87 | 1.15, 3.05 | 0.012 |
| **Disease Duration, days** |  |  |  |  |  |  |  |  |  |  |
| <30 | 62 | 8 | — | — |  | 62 | 8 | — | — |  |
| >=30 & <90 | 178 | 36 | 1.71 | 0.75, 3.92 | 0.203 | 178 | 36 | 2.34 | 0.97, 5.67 | 0.059 |
| >=90 | 105 | 28 | 2.45 | 1.04, 5.80 | 0.041 | 105 | 28 | 3.92 | 1.49, 10.29 | 0.006 |
| **Location** |  |  |  |  |  |  |  |  |  |  |
| Trunk | 187 | 36 | — | — |  |  |  |  |  |  |
| Head & Face | 51 | 9 | 0.90 | 0.40, 2.01 | 0.795 |  |  |  |  |  |
| Limbs | 107 | 27 | 1.42 | 0.80, 2.50 | 0.230 |  |  |  |  |  |
| **Pre-op NRS score** |  |  |  |  |  |  |  |  |  |  |
| <7 | 104 | 13 | — | — |  | 104 | 13 | — | — |  |
| >=7 | 241 | 59 | 2.27 | 1.18, 4.35 | 0.014 | 241 | 59 | 2.79 | 1.39, 5.58 | 0.004 |
| **Interventional procedure** |  |  |  |  |  |  |  |  |  |  |
| PRF | 197 | 38 | — | — |  | 197 | 38 | — | — |  |
| RF-TC | 76 | 17 | 1.21 | 0.63, 2.30 | 0.570 | 76 | 17 | 0.77 | 0.37, 1.59 | 0.482 |
| NES | 72 | 17 | 1.29 | 0.68, 2.47 | 0.437 | 72 | 17 | 1.11 | 0.55, 2.22 | 0.776 |
| Abbreviations: CI = Confidence Interval, OR = Odds Ratio | | | | | | | | | | |

Null deviance = 353; Null df = 344; Log-likelihood = -166; AIC = 353; BIC = 395; Deviance = 331; Residual df = 334; No. Obs. = 345

**Table S5** Association between TyG and Pain Prognosis with multiple regression models**(For the sensitivity analysis, poor pain prognosis at 3 months was defined solely as an NRS score ≥4)**

| **Characteristic** | **Model 1** | | | **Model 2** | | | **Model 3** | | |
| --- | --- | --- | --- | --- | --- | --- | --- | --- | --- |
|  | **OR** | **95% CI** | **P-value** | **OR** | **95% CI** | **P-value** | **OR** | **95% CI** | **P-value** |
| **TyG (continuous)** | 1.66 | 1.07, 2.56 | 0.023 | 1.97 | 1.23, 3.14 | 0.005 | 1.87 | 1.15, 3.05 | 0.012 |
| **TyG** |  |  |  |  |  |  |  |  |  |
| Q1 | — | — |  | — | — |  | — | — |  |
| Q2 | 1.28 | 0.58, 2.86 | 0.542 | 1.24 | 0.55, 2.81 | 0.600 | 1.21 | 0.53, 2.75 | 0.651 |
| Q3 | 1.59 | 0.73, 3.47 | 0.242 | 1.51 | 0.68, 3.34 | 0.313 | 1.42 | 0.63, 3.21 | 0.398 |
| Q4 | 2.14 | 1.01, 4.55 | 0.048 | 2.52 | 1.15, 5.50 | 0.021 | 2.26 | 1.00, 5.09 | 0.049 |
| P for trend |  |  | 0.038 |  |  | 0.017 |  |  | 0.044 |
| Model 1: no covariates were adjusted  Model 2: adjusted for Disease Duration, and Pre-op NRS score  Model 3: adjusted for Gender, BMI, Age, Diabetes, Interventional procedure, Disease Duration, and Pre-op NRS score | | | | | | | | | |

**Table S6.** RCS Model Diagnostics

a. RCS Model Coefficients.

| Characteristics | Coefficient | SE | Statistic | P-value |
| --- | --- | --- | --- | --- |
| Intercept | -9.847 | 8.798 | -1.119 | 0.263 |
| TyG | 0.800 | 1.078 | 0.742 | 0.458 |
| TyG' | -0.542 | 3.581 | -0.151 | 0.880 |
| TyG'' | 1.999 | 11.923 | 0.168 | 0.867 |
| Gender=1 | -0.055 | 0.269 | -0.203 | 0.839 |
| Age..years=1 | -0.268 | 0.288 | -0.931 | 0.352 |
| Interventional.procedure=2 | -0.010 | 0.342 | -0.028 | 0.978 |
| Interventional.procedure=3 | -0.036 | 0.348 | -0.103 | 0.918 |
| BMI | 0.011 | 0.043 | 0.265 | 0.791 |
| Duration..days=1 | 0.721 | 0.427 | 1.688 | 0.091 |
| Duration..days=2 | 1.412 | 0.466 | 3.032 | 0.002 |
| Diabetes=1 | 0.239 | 0.336 | 0.712 | 0.476 |
| aNRS.score=1 | 1.167 | 0.340 | 3.432 | <0.001 |

b. Model fit and multicollinearity diagnostics.

| Obs | Residual d.f. | d.f. | Model L.R. | P | g |
| --- | --- | --- | --- | --- | --- |
| 345 | 332 | 12 | 31.7896 | 0.0015 | 0.8794 |

**Table S7.** Subgroup analyses of the association between TyG index and pain prognosis.

| Subgroup | N | Adjusted OR (95% CI) | P value | P for interaction |
| --- | --- | --- | --- | --- |
| Overall | 345 | 1.99 (1.25-3.18) | 0.004 |  |
| Age, years |  |  |  | 0.566 |
| <=65 | 122 | 2.14 (1.01-4.55) | 0.048 |  |
| >65 | 223 | 1.77 (0.96-3.26) | 0.069 |  |
| Interventional procedure |  |  |  | 0.559 |
| PRF | 197 | 2.06 (1.07-3.95) | 0.030 |  |
| RF-TC | 76 | 1.11 (0.47-2.64) | 0.811 |  |
| NES | 72 | 4.65 (1.14-18.89) | 0.032 |  |
| Disease Duration, days |  |  |  | 0.524 |
| <30 | 62 | 3.46 (1.04-11.57) | 0.044 |  |
| >=30 & <90 | 178 | 1.46 (0.69-3.09) | 0.321 |  |
| >=90 | 105 | 2.43 (1.08-5.45) | 0.032 |  |
| NRS score |  |  |  | 0.444 |
| <7 | 104 | 3.24 (1.04-10.10) | 0.042 |  |
| >=7 | 241 | 1.82 (1.06-3.13) | 0.031 |  |
| Location |  |  |  | 0.635 |
| Trunk | 187 | 1.85 (0.94-3.64) | 0.075 |  |
| Head & Face | 51 | 2.76 (0.36-20.96) | 0.327 |  |
| Limbs | 107 | 3.02 (1.30-7.00) | 0.010 |  |
| Diabetes |  |  |  | 0.976 |
| No | 280 | 2.06 (1.20-3.52) | 0.008 |  |
| Yes | 65 | 1.61 (0.57-4.56) | 0.371 |  |
| Models were adjusted for gender, BMI, age, diabetes mellitus, intervention type, disease duration, and baseline NRS score, excluding the corresponding stratifying variable when it was part of this adjustment set. Models stratified by pain location retained all seven covariates. Interaction P values were obtained from full-cohort models including continuous TyG, the subgroup variable, their interaction term(s), and the remaining adjustment covariates. | | | | |
